# Stability of Coinage Metals Interacting with C_60_

**DOI:** 10.3390/nano9101484

**Published:** 2019-10-18

**Authors:** Navaratnarajah Kuganathan, Ratnasothy Srikaran, Alexander Chroneos

**Affiliations:** 1 Department of Materials, Imperial College London, London SW7 2AZ, UK; alexander.chroneos@imperial.ac.uk; 2 Faculty of Engineering, Environment and Computing, Coventry University, Priory Street, Coventry CV1 5FB, UK; 3 Department of Chemistry, University of Jaffna, Sir. Pon Ramanathan Road, Thirunelvely, Jaffna 40000, Srilanka; ratnasothysrikaran@gmail.com

**Keywords:** C_60_, copper, silver, gold, encapsulation energy

## Abstract

Buckminsterfullerene (C_60_) has been advocated as a perfect candidate material for the encapsulation and adsorption of a variety of metals and the resultant metallofullerenes have been considered for the use in different scientific, technological and medical areas. Using spin-polarized density functional theory together with dispersion correction, we examine the stability and electronic structures of endohedral and exohedral complexes formed between coinage metals (Cu, Ag and Au) and both non-defective and defective C_60_. Encapsulation is exoergic in both forms of C_60_ and their encapsulation energies are almost the same. Exohedral adsorption of all three metals is stronger than that of endohedral encapsulation in the non-defective C_60_. Structures and the stability of atoms interacting with an outer surface of a defective C_60_ are also discussed. As the atoms are stable both inside and outside the C_60_, the resultant complexes can be of interest in different scientific and medical fields. Furthermore, all complexes exhibit magnetic moments, inferring that they can be used as spintronic materials.

## 1. Introduction

Buckminsterfullerene (C_60_) is one of the allotropes of carbon exhibiting nanosized molecular structure with potential applications in chemistry, catalysis, material science, biology and medicine [1,2,3,4,5,6,7,8]. Encapsulation (atom located inside) and adsorption (atom located outside) of metal atoms have received great experimental and theoretical attention recently in order to optimise the application of C_60_, mainly in catalytic processes, and electronic and metal storage devices [1,2,3,4,9,10,11,12,13,14,15].

A variety of metal atoms have been encapsulated within C_60_ experimentally for different applications [1,2,3,4,5,6]. The preparation of endohedral fullerenes can be mainly carried out by inserting metals, either during the preparation of fullerenes in the arc-vaporization technique [16] or after the preparation of fullerenes through five- or six-membered rings [17]. The latter method requires a high kinetic energy barrier as guest atoms need to travel through the five- or six-membered rings. There are other experimental methods available in the literature for the encapsulation [18,19,20]. A laser vaporization technique was used to encapsulate La by Klinger et al. [12] and the electronic behavior of the resultant compound was investigated using tunneling conductivity measurements. A recoil implantation technique was applied to encapsulate radioactive isotopes including ^86^Zr and ^168^Hf [17]. The resultant radioactive complexes were suggested for future applications in medical science as direct contact of toxic guest atoms with biological systems in the body can be avoided. Electronic structure calculations based on density functional theory (DFT) on several endohedral fullerenes have been reported in the literature [21,22,23,24]. Recently, we studied the thermodynamical stability and electronic structures of volatile fission atoms (Xe, Kr, Br, I, Te, Rb and Cs) encapsulated within C_60_ using DFT simulation to predict the efficacy of C_60_ as a filter material in the spent fuel reprocessing [25].

Although there are many experimental studies available on endohedral fullerenes, only few experimental investigations have been reported on exohedral fullerenes [26,27,28]. Nevertheless, numerous theoretical simulations have been performed to study interactions of metal atoms on the surface of C_60_ [29,30,31,32]. Exohedral adsorption of a single atom and multiple atoms were studied by Sankar De et al. [29] using ab initio simulation in the absence of dispersion correction. In a very recent DFT simulation, we have shown the importance of dispersion correction for a Cd atom interacting with a C_60_ surface [33].

Encapsulation of coinage metals by C_60_ is of great interest as the resultant endohedral compounds can provide useful information about electronic structures of the encapsulated atoms required for the development of electronic materials. Huang et al. [34] synthesized Cu@C_60_ and its magnetic structure exhibits ring-current-induced magnetism. Experimental formation of Ag@C_60_ was reported by Narwade et al. [35] and its efficient electrocatalytic activity was tested in the fuel cell reaction. The growth of Au nanoparticles embedded in the C_60_ cage was experimentally characterised using a thermal co-evaporation technique by Singhal et al. [36]. Although there is an experimental interest in the literature, there are only few theoretical simulations of coinage metals interacting with C_60_.

In the present study, we used DFT with dispersion correction to examine the stability of coinage metal atoms interacting (Cu, Ag and Au) both inside and outside non-defective and defective C_60_ molecules. The current simulation method enables the examination of the electronic structures, charge transfer and magnetic moment of the composites. 

## 2. Computational Methods 

All calculations were based on the DFT. The VASP code [37,38] which solves the Kohn–Sham (KS) equations using plane wave basis sets was used. The exchange correlation term was modelled using the generalized gradient approximation (GGA) with the Perdew, Burke and Ernzerhof (PBE) function [39]. A plane wave basis set with a cut-off value of 500 eV was used in all cases. Energy minimisation was performed using the conjugate gradient algorithm [40] with a force tolerance of 0.001 eV/Å. A single k-point (Γ) point was used in all calculations to represent the Brillouin zone due to the large supercell. Coinage bulk structures were optimised using a 16 × 16 × 16 Monkhorst k-point mesh [41] which yielded 64 k points. Cubic supercells with 20 Å were used in all calculations to ensure that the two adjacent images do not interact with each other. Encapsulation and adsorption energies were calculated using the following equation:

(1) $$\mathrm{Eenc}/\mathrm{ads}=E\;\left(M–C_{60}\right)-E\;\left(C_{60}\right)-E\;\left(M\right),$$

where E(C_60_) is the total energy of non-defective or defective C_60_ molecule, E(M–C_60_) is the total energy of a metal atom interacting with a non-defective or defective C_60_ and E (M) is the total energy of an isolated metal atom. Here, dispersive interaction was included by using the pair-wise force field as implemented by Grimme et al. [42] in the VASP package.

## 3. Results 

### 3.1. Initial Configurations

**Figure 1:**
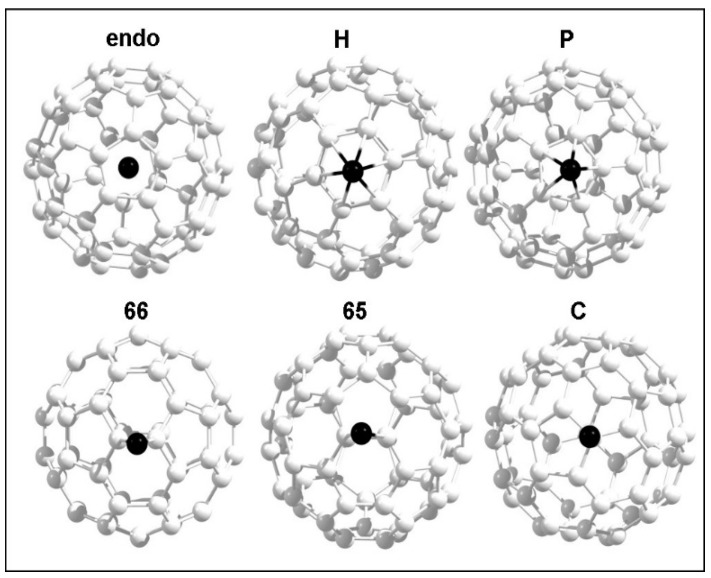
Initial configurations of a single metal atom interacting with a C_60_ molecule.

Six different positions were considered for the interaction of coinage metals with C_60_. At the endohedral position (endo), an atom occupies the center of a C_60_ molecule (refer to Figure 1). There are five different positions available on the outer surface of the C_60_ molecule for the adsorption of atoms, as shown in Figure 1. Positions “hex” and “pent” exhibit that the atom is just above the center of the hexagonal and pentagonal rings of the C_60_, respectively. In the 66 and 65 configurations, the atom sits on the C−C bond, connecting two adjacent hexagonal rings and connecting one hexagonal and its nearest neighbor pentagonal ring, respectively. In the configuration “C”, the metal atom is adsorbed by a C atom in the C_60_ cage.

### 3.2. Validation of the Pseudopotentials and Basis Sets 

In order to validate the choice of pseudopotentials and basis sets used in this study for C, Cu, Ag and Au, geometry optimisations were performed on a C_60_ molecule and bulk structures of coinage metals. Calculated geometrical parameters and electronic structures were then compared with corresponding experimental values and the values reported in other theoretical studies. 

#### 3.2.1. Calculation on Band Gap of a C_60_ Molecule

**Figure 2:**
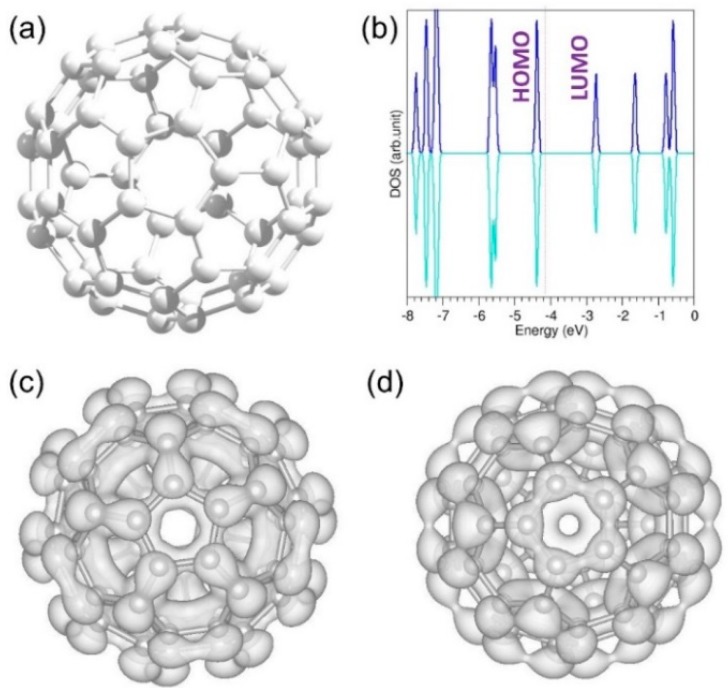
Relaxed structure (**a**), total density of state (DOS) (**b**), highest occupied molecular orbital (HOMO) (**c**), and lowest occupied molecular orbital (LUMO) (**d**) of a C_60_ molecule.

Geometry optimisation of a C_60_ molecule revealed bond length values of 1.45 Å and 1.40 Å for C−C and C=C, respectively. These values are in good agreement with the respective values of 1.43 Å and 1.39 Å reported experimentally [43]. The band gap was calculated by plotting the density of states (DOSs) and measuring the distance between the highest occupied molecular orbital (HOMO) and the lowest unoccupied molecular orbital (LUMO), as shown in the Figure 2. The estimated band gap value of 1.55 eV agrees well with the theoretical value of 1.64 eV [44]. Figure 2 shows the optimised structure of a C_60_ molecule together with its total DOS plot, HOMO and LUMO.

#### 3.2.2. Calculations of Energy Minimisation on Bulk Cu, Ag and Au

**Figure 3:**
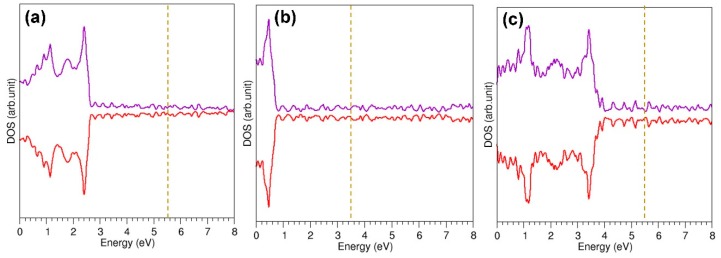
DOS plots for the bulk structures of Cu (**a**), Ag (**b**) and Au (**c**). Dotted lines correspond to the Fermi energy.

Energy minimisation calculations were performed on bulk Cu, Ag and Au (*fcc* structure) structures to obtain equilibrium lattice constants and cohesive energies. Cohesive energy was calculated by considering the energy difference between an isolated gas-phase atom and an atom in the bulk. The calculated equilibrium lattice constants and cohesive energies (refer to Table 1) are in good agreement with the experimental and calculated values. Zero magnetic moments were calculated for all three bulk structures. DOS plots for the bulk structures of Cu, Ag and Au (refer to Figure 3) confirm the non-magnetic nature of bulk coinage metals. Considerable progress has recently been made on accurate theoretical determination of electronic band structure of solids. In order to describe the band structure of bulk copper, self-energy of d electrons was included in first-principles GW calculations by Marini et al. [45] and good agreement between experiment and theory was observed. In another theoretical study by Goraus et al. [46], on-site Coulomb interaction (U) was included for Ag 4d states in CeAgGa and the calculated density of states was in satisfactory agreement with X-ray photoemission spectra.

### 3.3. Encapsulation of Coinage Metals within C_60_

First, the stability of a single coinage metal occupying the center of the cage was considered. Relaxed structures revealed that the encapsulated atoms (Cu, Ag and Au) are still in the center of the cage without altering their atomic positions. Encapsulation energy was calculated to determine the thermodynamical stability of the atoms when they are inside the C_60_. The equation showing the methodology of calculating encapsulation energy was reported in the methodology section. Calculations were carried out with and without dispersion. Encapsulation energies calculated using dispersion are exoergic, meaning that they are stable inside the C_60_ (refer to Table 2). Endoergic encapsulation is observed in calculations without dispersion, inferring the importance of the dispersion. Enhancement in the encapsulation is due to the additional attractive term introduced by the dispersion. The rest of the calculations in this study were only performed with dispersion.

Both Cu and Au have similar encapsulation energies. This can be due to the identical empirical atomic radii of Cu and Au (1.35 Å) although their calculated values are 1.45 Å and 1.74 Å, respectively. The calculated atomic radiis of Cu, Ag and Au are in ascending order. However, the encapsulation energy does not follow any trend with it. Bader charge analysis [49] shows that there is a small amount of charge transferred from metal atoms to the C_60_ cage. The outer electronic configuration of all three metal atoms is d^10^s^1^, meaning that the magnetic moment is one as there is an un-paired electron in the *s*-shell. The net magnetic moment of a C_60_ molecule is calculated to be zero. The magnetic moment of the resultant complex is ~1. This indicates that the electronic configuration of the metal is unaltered. This is further supported by the very small positive Bader charge on the metal atoms. 

**Figure 4:**
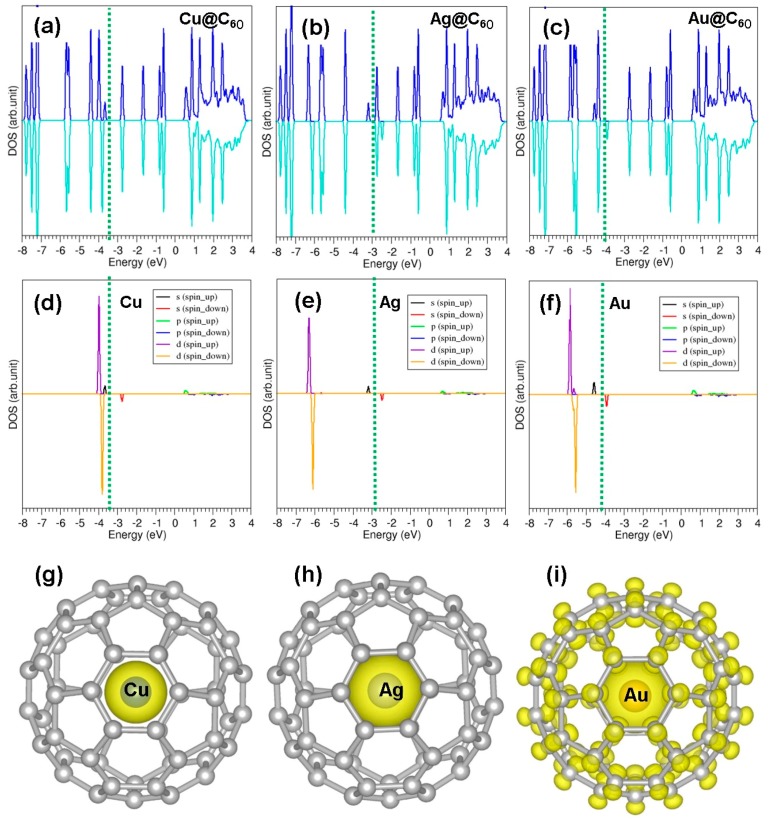
(**a**–**c**) Total DOSs for Cu, Ag and Au within a C_60_ molecule, respectively; (**d**–**f**) atomic doses for Cu, Ag and Au within a C_60_ molecule, respectively; and (**g**–**i**) charge density distributions around the encapsulated metal atoms for Cu, Ag and Au within a C_60_ molecule, respectively.

Figure 4 shows the calculated total and atomic DOS plots and partial charge density distribution around the encapsulated atoms. Encapsulation introduces extra peaks associated with *s* and *d* orbitals. In the case of Cu, there is a slight overlap between s and d states near the Fermi energy. The d states are further away (~3 eV) from the Fermi energy level while the s states are closer to it. Encapsulation of Au introduces the s states near the Fermi level and the d states are 2 eV away from it.

### 3.4. Adsorption of Coinage Metals on the Surface of C_60_

Here, we considered the adsorption of metal atoms by the outer surface of C_60_. As discussed above, five different initial configurations were considered. Table 3 reports the final configurations and relative adsorption energies (refer to the methodology section for the equation that was used to calculate the adsorption energy) with respect to the most stable configuration. 

**Figure 5:**
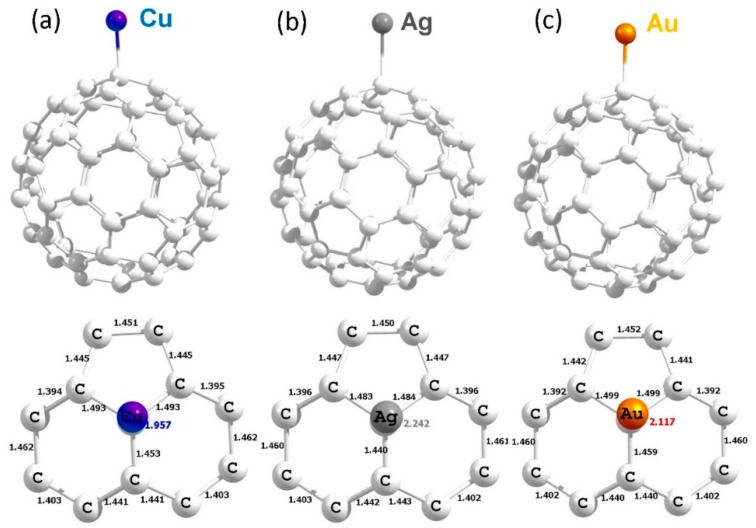
Optimised structures of Cu (**a**), Ag (**b**) and Au (**b**) adsorbed on the surface of C_60_.

The configuration “**C**” was found to be the most stable adsorption site and this is in agreement with the theoretical study reported by Shankar et al. [29]. Figure 5 shows the most stable geometries and bond distances formed between metal atoms and the C_60_. Calculated adsorption energies are negative, meaning that all three metals are stable on the surface of the C_60_. Copper forms a shorter Cu–C bond distance of 1.957 Å than the Ag–C_60_ and Au–C_60_ bond distances, which reflects in the adsorption energy and the Bader charge (refer to Table 4). The C–Ag bond distance is 2.242 Å which is slightly longer than the C–Cu and C–Au bond distances. The lower adsorption energy can be attributed to the longer C–Ag bond distance. In the case of Au, the adsorption energy is 0.09 eV less than that calculated for Cu. This is evidenced by the intermediate Au–C bond distance of 2.117 Å. Magnetic moments are not altered significantly. However, Bader charge and magnetic moment values are slightly higher and lower than that found in the encapsulated complexes, respectively, confirming the adsorption nature of atoms is stronger than encapsulation.

**Figure 6:**
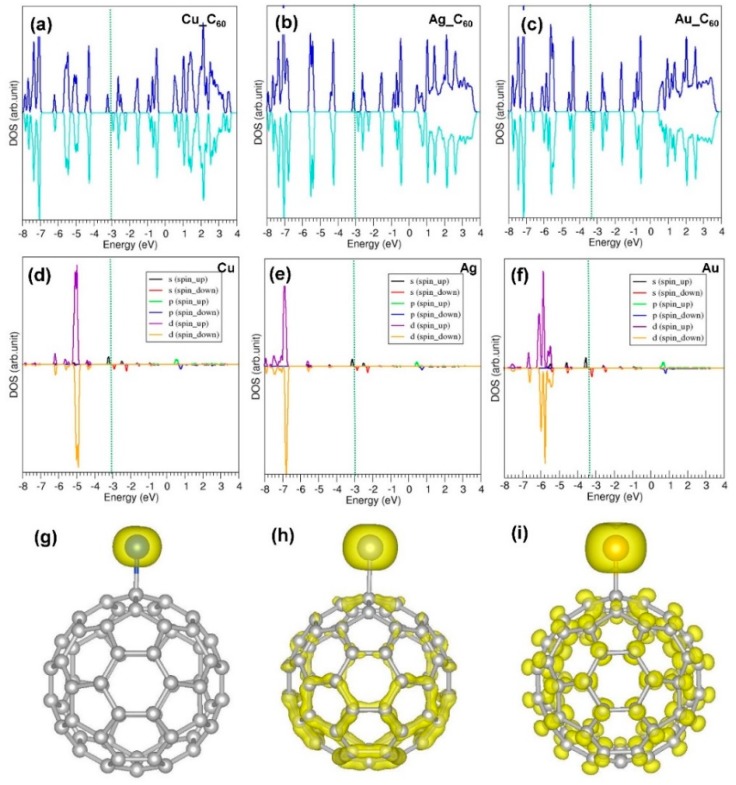
(**a**–**c**) Total DOSs for Cu, Ag and Au, respectively; (**d**–**f**) atomic doses for Cu, Ag and Au, respectively; and (**g**–**i**) charge density distributions around the adsorbed metal atoms for Cu, Ag and Au, respectively.

Figure 6 shows the total DOSs, atomic DOSs and charge density distribution plots showing the interaction of atoms with C_60_. Additional states arising from *s* and *d* orbitals are introduced between the top of the valence band and bottom of the conduction band without altering the band gap significantly.

### 3.5. Defective C_60_ Structure

**Figure 7:**
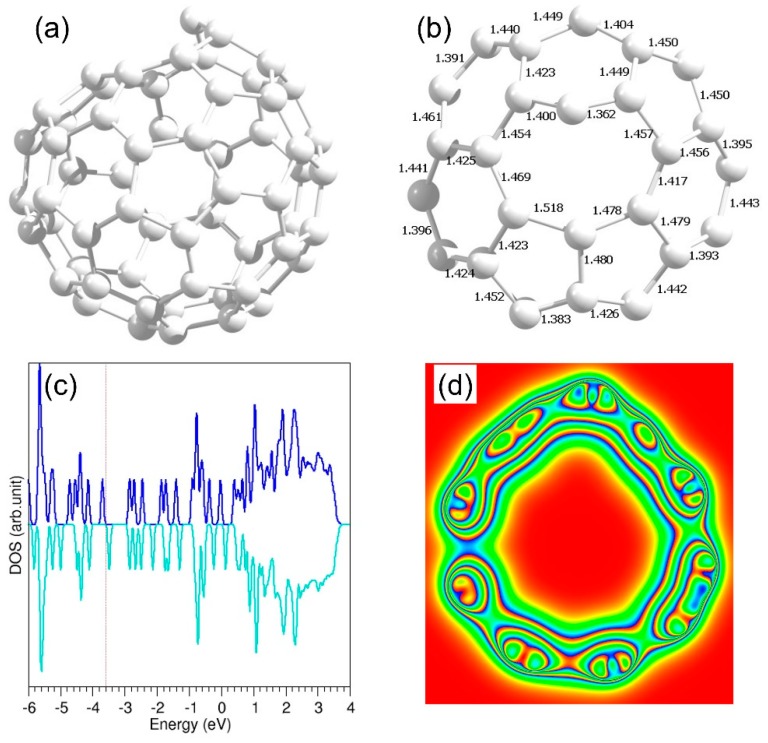
(**a**) Relaxed structure of a defective C_60_ molecule (**b**) bond distances around the defect (**c**) DOS and (**d**) cross sectional charge density plot.

Next, we considered a defective C_60_ structure to examine the encapsulation or adsorption capability of coinage metal atoms. A defect was introduced in C_60_ by removing a C atom. The relaxed structure, and DOS and charge density plots of defective C_60_ are shown in Figure 7. In previous experimental and theoretical studies, non-defective or defective single-walled nanotubes and C_60_ have been considered for the reaction with transition metals, molecules, one-dimensional crystals and metal clusters [52,53,54,55,56,57]. 

The defective C_60_ structure forms an eight-membered ring with distorted C–C bond distances. The C_60_ molecule has now lost its symmetry and the outer surface of the optimised structure is expected to be more reactive with metal atoms. Furthermore, encapsulation via the eight-membered ring with larger open space can be easier than either a six- or a five-membered ring. 

### 3.6. Encapsulation of Metal Atoms within a Defective C_60_ Molecule 

**Figure 8:**
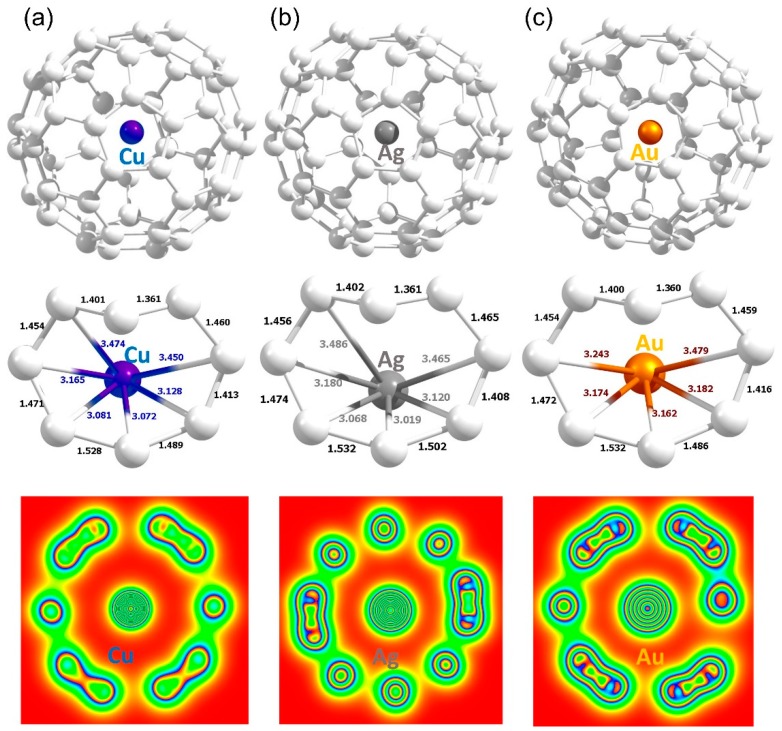
Relaxed structures and charge density plots of Cu (**a**), Ag (**b**) and Au (**c**) encapsulated within a defective C_60_ molecule.

In the optimised structures, the positions of the atoms are slightly deviated from the center of the cage and towards the defective part of the C_60_ molecule (refer to Figure 8). This is because of the non-symmetrical nature of the defective C_60_ molecule. The calculations show that the encapsulation energies are exoergic, meaning that they are more stable in the cage of defective C_60_ molecule than in isolated atoms (refer to Table 5). Encapsulation energies are approximately −0.50 eV in all cases, indicating that interactions between metal atoms and the defective C_60_ molecule are almost the same. This is further supported by the similar C‒M bond distances in the relaxed structures (refer to Figure 8). Bader charge analysis shows that metal atoms transfer a small amount of charge to C_60_. The magnetic moment of the defective C_60_ molecule is zero. In all cases, magnetic moments of complexes are one, meaning that electronic configurations of coinage metal atoms are almost unaltered.

### 3.7. Adsorption of Metal Atoms on the Surface of Defective C_60_ Molecule 

**Figure 9:**
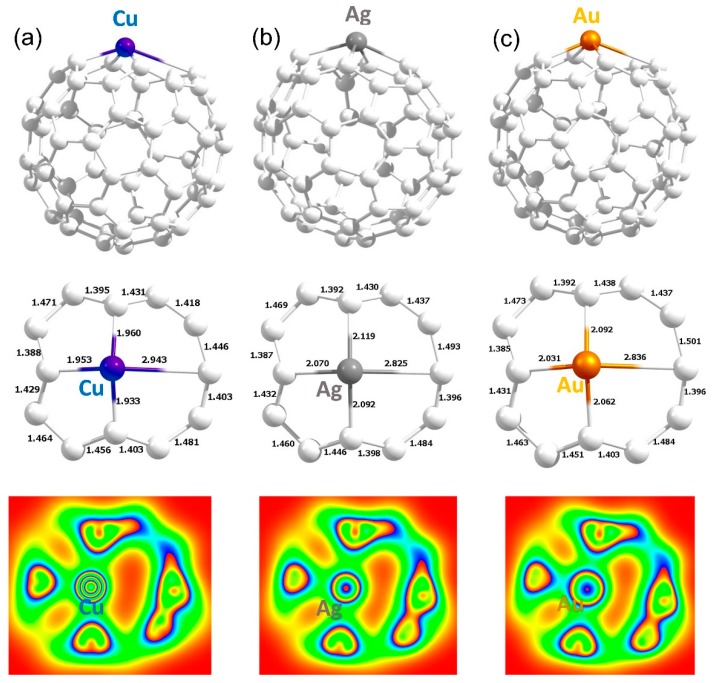
Relaxed structures and charge density plots of Cu (**a**), Ag (**b**) and Au (**c**) adsorbed on the surface of defective C_60_ molecule.

Finally, interactions between atoms and defective surface of C_60_ were considered. There is a strong interaction between defect and metal atoms (refer to Figure 9). Metal atoms occupy the vacant side forming strong bonds with adjacent carbon atoms. This is evidenced by the bond distances, the amount of charge transferred (refer to Table 6), charge density plots and the reduction in the magnetic moments.

## 4. Conclusions

In this study, DFT simulations, together with dispersion correction, were performed to examine the encapsulation and adsorption capability of both non-defective and defective C_60_ molecules. Calculations show that the non-defective C_60_ can trap all three metals both endohedrally and exohedrally. Significant enhancement is observed for the exohedral adsorption compared to for the endohedral encapsulation in the non-defective C_60_. Furthermore, the defective C_60_ was examined for trapping both endohedrally and exohedrally. Encapsulation and adsorption energies are exoergic, suggesting that the defective C_60_ is also a candidate host material for trapping coinage metals. Both non-defective and defective C_60_ structures can be ideal host materials in scientific and medical fields. As resultant M–C_60_ complexes are magnetic, they can play an important role in spintronic devices.

## Figures and Tables

**Table 1:** Calculated and experimental lattice constants and cohesive energies of bulk coinage metals.

	Parameter					
	Lattice Constant (Å)			Cohesive Energy (eV)		
	Cu	Ag	Au	Cu	Ag	Au
Proposed method	3.57	4.09	4.12	3.99	2.97	3.63
Experiment	3.59 [47]	4.06 [47]	4.06 [47]	3.48 [48]	2.94 [48]	3.81 [48]
Other theory	3.501–3.686 [48]	4.046–4.321 [48]	4.084–4.112 [48]	2.54–4.42 [48]	1.87–3.60 [48]	2.23–3.86 [48]

**Table 2:** Encapsulation energies of single coinage metal atoms incorporating the C_60_ molecule, Bader charges on metal atoms and magnetic moments of the resultant composites.

System	Atomic Radius (Å)		Encapsulation Energy (eV)		Bader Charge |e|		Magnetic Moment (µ)	
	Empirical [50]	Calculated [51]	DFT	DFT + D	DFT	DFT + D	DFT	DFT + D
Cu@C_60_	1.35	1.45	0.13	**−0.58**	+0.1675	+0.1672	1.0000	1.0000
Ag@C_60_	1.60	1.65	0.32	**−0.36**	+0.1704	+0.1701	0.9983	0.9983
Au@C_60_	1.35	1.74	0.23	**−0.56**	+0.0956	+0.0954	0.9985	0.9985

**Table 3:** Initial and final configurations of coinage metal atoms adsorbed by the outer surface of the C_60_.

Initial Configuration	Final Configuration and Relative Adsorption Energies (eV)		
	Cu	Ag	Au
H	C (0.00)	H (0.33)	H (0.67)
P	P (0.47)	P (0.30)	P (0.69)
66	66 (0.12)	C (0.00)	C (0.00)
65	65 (0.02)	65 (0.07)	C (0.00)
C	C (0.00)	C (0.00)	C (0.00)

**Table 4:** Adsorption energies of atoms interacting with the surface of C_60_, Bader charge on the metal atoms and magnetic moments of the composites.

System	Adsorption Energy (eV)	Bader Charge |e|	Magnetic Moment (µ)
Cu_C_60_	−0.98	+0.30	0.9711
Ag_C_60_	−0.50	+0.24	0.9329
Au_C_60_	−0.89	+0.32	0.9778

**Table 5:** Encapsulation energies, Bader charge on the metal atoms and magnetic moments of the endohedral composites formed between the defective C_60_ molecule and metals.

System	Encapsulation Energy (eV)	Bader Charge |e|	Magnetic Moment (µ)
Cu@C_60_defe_	−0.56	+0.27	1.000
Ag@C_60_defe_	−0.43	+0.49	1.000
Au@C_60_defe_	−0.51	+0.31	1.000

**Table 6:** Encapsulation energies, Bader charge on the metal atoms and magnetic moments of the composites formed between the surface of defective C_60_ molecule and metals.

System	Encapsulation Energy (eV)	Bader Charge |e|	Magnetic Moment (µ)
Cu_C_60_defe_	−0.61	+0.70	0.8896
Ag_C_60_defe_	−0.41	+0.62	0.9012
Au_C_60_defe_	−0.48	+0.51	0.9414
